# Immune Response against ALK in Children with ALK-Positive Anaplastic Large Cell Lymphoma

**DOI:** 10.3390/cancers10040114

**Published:** 2018-04-10

**Authors:** Serena Stadler, Vijay Kumar Singh, Fabian Knörr, Christine Damm-Welk, Wilhelm Woessmann

**Affiliations:** Department of Pediatric Hematology and Oncology, Justus-Liebig University, D-35392 Giessen, Germany; serena.stadler@paediat.med.uni-giessen.de (S.S.); vijay.singh@paediat.med.uni-giessen.de (V.K.S.); fabian.knoerr@paediat.med.uni-giessen.de (F.K.); christine.damm-welk@paediat.med.uni-giessen.de (C.D.-W.)

**Keywords:** anaplastic large cell lymphoma, ALK, ALK-positive ALCL, immune response, ALK-specific T cells, ALK autoantibodies

## Abstract

Patients with anaplastic lymphoma kinase (ALK)-positive anaplastic large cell lymphoma (ALCL) mount a humoral and cellular immune response against ALK. More than 90% of children and adolescents with ALK-positive ALCL have detectable anti-ALK antibodies in serum or plasma, and the antibody titer inversely correlates with the risk of relapse. ALK-specific CD8 and CD4 T cell responses have been described in patients with ALK-positive ALCL. Vaccination with ALK DNA led to protection against lymphoma growth in a murine model. Collectively, these data suggest that the ALK-specific immune response is involved in the control of the disease. The characteristics of the humoral and cellular immune response against ALK as well as tumor immune escape mechanisms have been increasingly investigated. However, tumor and host factors contributing to the individual immune response against ALK are still largely unknown. Depending on the individual strength of the immune response and its determinants, individualized immunological approaches might be appropriate for the consolidation of ALCL patients. Strategies such as ALK vaccination could be effective for those with a pre-existing anti-tumor immunity, while an allogeneic blood stem cell transplantation or check-point inhibition could be effective for others.

## 1. Introduction

Anaplastic lymphoma kinase (ALK)-positive anaplastic large cell lymphoma (ALCL) is a biologically defined disease of children and young adults [1]. It accounts for 10–15% of pediatric and adolescent non-Hodgkin lymphomas [2]. As a T cell lymphoma, defined by so-called hallmark cells and the expression of CD30, ALCL is categorized in several histological subtypes. ALK-positive ALCL is distinguished from ALK-negative systemic ALCL and CD30-positive cutaneous lymphoproliferations by the expression of ALK. The latter two entities are exceedingly rare in children. Almost 90% of ALK-positive ALCLs in children carry a characteristic t(2;5) (p23;q35) chromosomal translocation, leading to the intracellular expression of the oncogenic fusion protein nucleophosmin (NPM)-ALK [3,4,5]. ALK fusion proteins are constitutively active tyrosine kinases with an essential role in lymphomagenesis and tumor survival [6,7,8]. 

Clinically, ALK-positive ALCL is characterized by a high incidence of B-symptoms (60%) and extra-nodal involvement (60%), particularly in the skin, lung, bone, and soft tissue [9,10,11,12,13,14,15,16,17]. Almost 90% of cases manifest as nodal disease, but extra-nodal and general symptoms are often concomitant, mimicking other diseases, such as pneumonia, chronic infections, autoimmune disease, or bone tumors. Current standard therapies for ALCL in children and adolescents, most commonly based on short-pulse chemotherapy courses, reach event-free survival rates of 70% [11,15,16,17,18]. Several new treatment options including ALK kinase inhibitors and anti-CD30 drug conjugates are now available, all of which have been shown to induce remissions in a high proportion of relapsed patients [19,20,21,22,23]. However, their role for long-term disease control or for the front-line treatment of ALCL patients still needs to be defined in clinical studies, especially since the induction of remission is not a surrogate marker for cure or long-term disease control in ALCL. Patients with relapsed ALK-positive ALCL still have a 60% survival chance with very different re-inductions and consolidations [24,25,26,27]. However, it is currently unclear which consolidation could be effective for individual relapsed patients. Long-term low-dose chemotherapy approaches, such as vinblastine monotherapy or allogeneic blood stem cell transplantation (SCT), might be potential options to treat these patients. 

**Figure 1:**
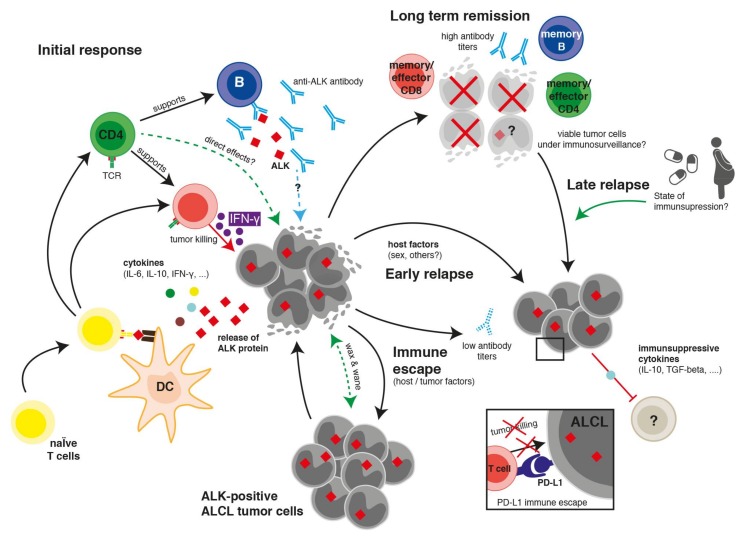
Current understanding of the immune response against anaplastic lymphoma kinase (ALK) in patients with ALK-positive anaplastic large cell lymphoma (ALCL). On the left, the primary anti-tumor response is shown. ALK (red squares) is processed and presented by dendritic cells (DCs) to cells of the adaptive immune system. Cytokines (small circles) and other factors shape the type of the immune response. Long-term remission is characterized by the presence of an immunologic memory. Putative mechanisms and factors that could lead to immune escape and relapse are shown in the lower right.

Wildtype ALK is a tyrosine kinase receptor belonging to the insulin receptor super family. During embryogenesis, ALK is highly expressed mainly in the nervous system but its presence in adult normal tissue is almost absent [3,28]. The limited expression of ALK in immune-privileged sites and the central role of ALK fusion proteins in the development and maintenance of ALCL suggest that ALK has the potential to serve as an attractive target for cancer immunotherapy. Studies on the immunogenicity of the ALK oncoprotein revealed both ALK-specific antibodies and T cell responses in ALK-positive ALCL patients. In this review, we summarize the current knowledge of the immune response against ALK in patients with ALK-positive ALCL (Figure 1) and the potential for the development of a clinical ALK-directed immunotherapy.

## 2. Clinical, Laboratory, and Pathological Hints towards an Immune Reaction against ALCL

Clinical, histopathological, and clinical laboratory observations provided the first hints that ALCL might provoke an immune response in patients.

More than 60% of children and adolescents with ALK-positive ALCL present with B-symptoms, suggesting an unspecific stimulation of the immune system by the lymphoma [11,16]. More direct clinical hints towards a possible involvement of the immune system in the control of ALCL have been observed even before the molecular pathogenesis of ALCL was discovered. A very peculiar observation is a so-called “wax and wane” course of the disease before final diagnosis in some patients. A clear lymphoma may disappear without therapy to grow again within weeks and sometimes months. These courses indicate that the immune system might initially control the lymphoma but finally fails. ALK-positive ALCL usually relapses within a few months after the end of initial therapy. However, very late relapses of ALK-positive ALCL between five and up to 20 years from initial diagnosis have also been observed. One could hypothesize that this reflects a weakening immunological control, especially since in some of these patients the disease reappeared during a time of immunosuppression such as pregnancy or treatment of an autoimmune disease [29].

The very low relapse rate of 10–20% in patients with progressive or relapsed ALCL after allogeneic SCT suggests that a graft versus ALCL effect may exist [30,31,32,33]. In contrast to almost all other lymphoid malignancies, allogeneic blood stem cell transplantation can be effective in ALK-positive ALCL patients with active disease [30,33]. 

Tumor cells in ALK-positive ALCL cells are often surrounded by abundant reactive bystander cells which suggest an accompanying immune reaction [34,35]. The amount of tumor cells in a biopsy might vary between less than 10% and more than 90%. Furthermore, the histological subtype of ALCL is independently associated with the risk of relapse [10,34,36] and the bystander infiltrate as well as the surface marker expression on ALCL cells differ according to the subtype [35]. These observations collectively hint towards a cellular immune interaction between bystander cells and tumor cells.

Pro-inflammatory cytokines are detected in the serum of ALCL patients at the time of diagnosis [37,38,39]. An ALCL-typical cytokine signature includes elevated levels of IL-9, IL-10, IL-17a, HGF, sIL-2R, and sCD30 [39]. Also, more than 10% of patients present with a macrophage activation or hemophagocytic syndrome, which is associated with a cytokine storm [11,16,40]. These observations indicate that ALCL is a cytokine-active lymphoma that induces at least an unspecific immune reaction in patients. Among ALK-positive ALCL patients, the serum concentration of the pro-inflammatory cytokines IL-6, IFN-γ, IP-10, and sIL-2R correlates with the clinical and biological characteristics as well as the risk of relapse. This suggests that ALCL cells might influence the immune system of patients, leading to a different outcome [39].

## 3. Humoral Immune Response against ALK

Humoral immune responses against tumor-associated antigens have been described in many cancers [41]. Autoantibody production against tumor antigens can be induced by mutated oncogenes, the emergence of neo-antigens, protein overexpression, the aberrant expression of proteins that are not expressed in normal tissue, the release of intracellular tumor antigens after inflammation or cell death, and aberrant post-translational modifications of proteins [42,43]. In 2000, Karen Pulford and colleagues investigated the humoral immune response against ALK in patients with ALCL. Using an indirect immunocytochemical approach, the authors detected circulating antibodies against ALK in the plasma of eleven ALK-positive ALCL patients but not in healthy controls [44]. The presence of anti-ALK antibodies in ALCL patients at different time points after diagnosis was confirmed in a subsequent analysis by the same group [45]. Mussolin et al. described anti-ALK antibodies in the sera of 25/28 pediatric ALK-positive ALCL patients [46]. Although statistically not significant, patients with higher ALK antibody titers prior to and after therapy had a trend toward a reduced relapse risk.

**Figure 2:**
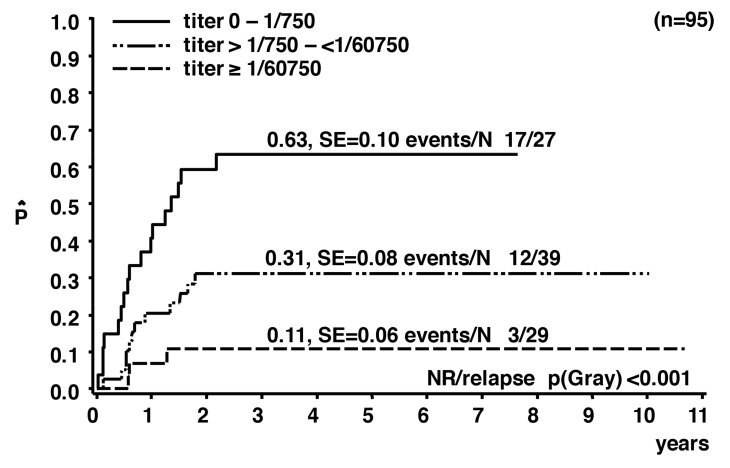
Cumulative incidence of relapse of patients with ALK-positive ALCL according to the anti-ALK antibody titer (adapted from Reference [47]).

The analysis of pretreatment anti-ALK antibodies in 95 ALK-positive pediatric ALCL patients, enrolled in clinical studies with comparable short-pulse chemotherapies, confirmed that more than 90% of the patients had measurable anti-ALK antibody titers at diagnosis compared to only one of 99 controls [47]. When categorizing patients’ anti-ALK antibody titers into low (≤1/750), intermediate (1/750 to <1/60,750) and high (≥1/60,750) titers, a significant correlation with clinical and biological risk factors was demonstrated. The anti-ALK antibody titers inversely correlated with the risk of relapse. The cumulative incidence of relapse was 11 ± 6% for patients in the high titer group compared to 31 ± 8% and 63 ± 10% for those in the intermediate and low titer groups, respectively (*p* < 0.001; Figure 2). If the antibody titers are regarded as a “readout” for the strength of the overall immune response against ALK, this correlation can be interpreted as an involvement of the patients’ immune response against ALK in the control of the disease. 

A systematic analysis of the course of anti-ALK antibody titers was performed during standard short-pulse chemotherapy in 122 pediatric NPM-ALK positive ALCL patients [48]. The persistence of the anti-ALK titer above 1/750 at the end of therapy as well as a moderate reduction of anti-ALK titers compared to pretreatment values predicted a protection against relapses [48]. The measurement of the ALK antibody titer may be useful as a prognostic parameter for risk stratification or as a surrogate marker for the measurement of the strength of patients’ ALK-specific immune response. Combining the biological risk factors, anti-ALK antibody titer and minimal disseminated disease, allowed the identification of pediatric NPM-ALK-positive ALCL patients with a very low risk of relapse [49]. 

The presence of anti-ALK antibodies was also observed in ALCL patients with variant ALK fusion partners, ALK-positive diffuse large B cell lymphoma, and in ALK-positive non-small cell lung carcinoma (NSCLC) [50,51]. Epitopes within the intracytoplasmic domain of ALK recognized by ALK autoantibodies were described in nine ALK-positive NSCLC patients [51]. Whether the strength of the humoral immune response against ALK is associated with the recognition of specific epitopes in the ALK protein needs to be further evaluated.

It is not clear so far whether ALK-specific autoantibodies may act against tumor cells or are merely a surrogate marker for the strength of the patients’ overall cellular immune response against ALCL. A direct anti-tumor effect of autoantibodies has been shown for antibodies against cell surface antigens. Examples include HER2 autoantibodies that suppress the activity of the HER2 receptor in some patients with HER2-positive breast or ovarian cancer after vaccination with HER2 specific peptides [52]. However, ALK fusion proteins are expressed exclusively intracellularly. Therefore, autoantibodies against ALK might not have direct anti-tumor activity but rather represent a surrogate marker for the ALK-specific T cell response. This is supported by the observation that the vaccination of B cell-deficient BALB/C mice with ALK plasmid DNA showed a protection against tumor growth and a cytotoxic T cell response after challenge with ALK-positive lymphoma cells [53]. 

## 4. Cellular Immune Response against ALK

ALK is spontaneously recognized as tumor antigen in ALK-positive ALCL patients. Several studies demonstrated the presence and persistence not only of an antibody response to ALK but also of ALK-specific CD8 and CD4 T cells in patients with ALK-positive ALCL.

### 4.1. CD8 T Cell Response against ALK

The immunogenic potential of ALK in initiating a cytotoxic T cell (CTL) response was first demonstrated in a study by Passoni et al. [54]. In a reverse immunological approach, two HLA-A*02:01-binding ALK-derived predicted peptides were tested for their capacity to initiate a specific CTL immune response in vivo in HLA-A*02:01 transgenic mice and in vitro in lymphocytes of HLA-*02-positive donors. Functional anti-ALK CD8 T cell precursors could be detected within the peripheral T cell repertoire of healthy donors. The generated donor-derived ALK-specific CTLs induced an antigen-specific HLA-A*02:01 restricted response with significant IFN-γ release. These cells were able to effectively kill HLA-matched ALCL and neuroblastoma cell lines endogenously expressing ALK [54]. These findings clearly identified ALK as a tumor antigen with the potential to elicit antigen-specific CD8 T cell responses.

A CTL response to ALK in ALK-positive ALCL patients was subsequently reported by Ait-Tahar et al. [45]. In this study, an IFN-γ ELISPOT analysis was used to detect CTL responses against two ALK-derived HLA-A*02:01-restricted peptides after the short-time culture of mononuclear cells of seven ALK-positive ALCL patients, two ALK-negative ALCL patients, and six healthy controls. A significant IFN-γ response was detected in the patients with ALK-positive ALCL but not in the controls. In two patients the ALK-specific response increased after weekly peptide re-stimulation and the generated CTLs successfully lysed ALCL cell lines or peptide pulsed cells in vitro in an MHC class I restricted manner [45]. Since the responsive patients were in clinical remission at the time of analysis, the anti-ALK CTL response after single stimulation indicates the presence of long-lived memory T cells with possible protective immunity. 

The natural frequency and functional phenotype of circulating anti-ALK CD8 T cell precursors in the peripheral blood of healthy donors and ALK-positive ALCL patients was assessed by tetrameric MHC/peptide analysis, IFN-γ ELISPOT assay, and in vitro lysis of ALK-positive target cells [55]. High frequencies of ALK-specific CD8 T cells were found in both patients and healthy donors. However, the immunological phenotype of anti-ALK CD8 T cells revealed effector and memory cells only in patients. In healthy donors, the CD8/ALK tetramer positive lymphocytes showed a predominantly naïve phenotype. To evaluate the functional potential of the memory cells and to investigate a potential secondary immune response, the CTLs were stimulated once with the ALK-derived peptide p280-89. The patients CTLs released IFN-γ and GM-CSF and successfully killed the ALK-positive ALCL cell lines, demonstrating their functional activity [55]. 

Memory CD8 T cells are required for protective anti-tumor immunity [56,57,58]. Reinforcing the immunological memory to ALK might, therefore, provide the basis for vaccination strategies. 

Chiarle et al. examined the in vivo potential and clinical relevance of vaccination with ALK cDNA against ALK-positive ALCL in mice [53]. BALB/c mice were vaccinated with plasmids encoding for the cytoplasmic portions of ALK and subsequently challenged with ALK-positive lymphoma cells. The immunization led to a long-lasting local and systemic lymphoma protection in ALK-vaccinated mice. The protection elicited ALK-specific IFN-γ responses and CD8 T cell-mediated cytotoxicity. The potential of the ALK vaccine was also assessed in a therapeutic setting. Mice were first challenged subcutaneously with lymphoma cells followed by vaccination. A significant protection against lymphoma growth was only detected in mice with limited tumor burden (up to 1 × 10^5^ cells). However, the combination of chemotherapy (a single dose of doxorubicin) with following ALK vaccination significantly enhanced the survival of mice challenged intravenously with 1 × 10^6^ ALK-positive lymphoma cells before therapy [53]. This study showed the potential of a vaccination against ALK in preventing lymphoma growth in vivo. Of note, a DNA-based ALK vaccination also showed a strong ALK-specific CTL immune response that inhibited primary tumor growth in grafted and primary mouse models of ALK-positive lung cancer [59]. The immunological targeting of ALK thus provides a potent therapeutic option to treat ALK-positive human cancers.

As a prerequisite for the clinical development of immunotherapeutic strategies, we recently examined NPM-ALK-specific CD8 T cell responses in NPM-ALK-positive ALCL patients in remission after chemotherapy [60]. To circumvent HLA-preselection and to ensure endogenous NPM-ALK peptide processing, we used autologous dendritic cells (DCs) transfected with in vitro transcribed NPM-ALK mRNA as antigen presenting cells (APCs) to stimulate donor-derived CD8 T cells. The stimulated CD8 T cells were then analyzed in an IFN-γ ELISPOT assay. Three out of five ALCL patients, but none of the healthy donors, had HLA-C-restricted CD8 T cell responses to NPM-ALK. One patient exhibited a CD8 T cell response after short-time stimulation, indicating a reactivation of persisting ALK-specific memory cells. Of note, the three patients with measurable ALK-specific CD8 T cells had a high anti-ALK antibody titer prior to therapy and a persistent titer at the time of analysis [60]. This confirms both sustained ALK-specific humoral and CD8 T cell responses in patients in clinical remission up to nine years after diagnosis.

Taken together, these in vitro and in vivo data reveal the existence of ALK-specific CD8 T cell responses in ALK-positive ALCL. The possible role of these CTLs in lymphoma protection, however, needs to be investigated in more detail.

### 4.2. CD4 T Cell Response against ALK

The presence of IgG antibodies and CD8 memory T cells against ALK in patients suggests the involvement of CD4 T cells in the anti-ALK immune response [61,62]. A study by Ait-Tahar et al. provided the first evidence of ALK-specific CD4 T cells in ALK-positive ALCL patients [63]. Using an IFN-γ ELISPOT assay, the authors showed that two in silico selected DRB1-restricted ALK-derived peptides were immunogenic in ALK-positive ALCL patients but not in ALK-negative ALCL patients or healthy controls. Mononuclear cells from all ALK-positive ALCL patients exhibited a significant IFN-γ response to the peptides, which could be intensified following repeated stimulation. The CD4 T cell-mediated and DRB1-restricted nature of the anti-ALK response was demonstrated by CD4 T cell depletion and the addition of anti-HLA-DR antibodies, which abrogated the IFN-γ release to both peptides. Peptide-specific CD4 T cell lines raised from one patient recognized and lysed ALK-positive tumor cell lines in an MHC class II restricted manner [63]. The majority of ALK-positive ALCL patients were in clinical remission and exhibited an antibody response to ALK at the time of analysis. Therefore, the presence of a significant CD4-mediated IFN-γ response suggests the existence of effector/memory CD4 Th1 subsets in ALK-positive ALCL patients. This might play an important role in protective tumor immunity as well as in the maintenance of the CTL memory response and the production of antibodies against ALK. 

The paucity of data regarding ALK-specific CD4 T cell response in ALK-positive ALCL patients demands further characterization of the CD4 T cell response in these patients.

## 5. Immune Escape Mechanisms

In cancer patients, a protective anti-tumor response is impaired. Cells of the immune system may not detect tumor antigens or recognize them as self rather than foreign. Also, immunosuppressive factors from the tumor microenvironment or the tumor itself might dampen tumor-reactive effector cells [64,65]. While the characteristics of the immune response against ALK as well as its possible clinical impact are increasingly unraveled, little is known about the factors influencing the inter-individual differences in the strength of the immune response. Currently, there are only a few hints regarding host factors influencing the immune response against ALK. The observation that more girls with ALCL mount a high antibody titer compared to boys indicates an influence of host factors in the immune response [47]. Patients with persistent ALK antibody titers at the end of chemotherapy have a reduced relapse risk compared to those with a low or no titer [48]. This observation indicates that the influence of chemotherapy on the immune response against ALK might depend on the individual patient’s immune system. Polymorphisms in genes involved in immunity are under investigation. Whether differences in the bystander infiltrate in ALCL are caused by differences in the host immune response or by different immune subversion modalities employed by the ALCL is not known. 

However, even if neoplastic cells are visible to the immune system, during carcinogenesis, cancer cells acquire complex immune escape mechanisms protecting themselves from immune surveillance. Some ALK-dependent immune escape mechanisms have been described for ALK-positive ALCL. A direct molecular link between ALK activation and suppression of the anti-tumor immune response has been shown by the Wasik group. In this study, it was demonstrated that NPM-ALK induces the expression of immunosuppressive programmed death-ligand 1 (PD-L1) in ALCL cells through STAT3 [66]. In another study, the inhibition of ALCL-associated allogeneic T cells could be experimentally reversed by PD-L1 blockage [67]. 

ALK-positive ALCL cell lines have been shown to secrete TGF-beta, IL-10 and express FoxP3 [68]. Elevated concentrations of IL-10 in the sera of children with ALK-positive ALCL before treatment correlated with the presence and quantity of circulating tumor cells [39]. It was shown that IL-10 decreases the expression of MHC class II molecules on monocytes or dendritic cells [69,70,71]. However, whether IL-10 secreted by ALK-positive tumor cells affects MHC class II expression in ALCL patients has not been shown directly. 

## 6. Therapeutic Implications

The increasing evidence for the existence and clinical relevance of an autologous immune response against ALK implies that immunotherapeutic approaches might be an effective therapeutic intervention for the treatment of ALK-positive ALCL patients.

Certain chemotherapeutic drugs exhibit immune stimulatory effects. By applying this knowledge, classical therapies could be further developed to a backbone with less acute and long-term toxicity. Even the current intensive polychemotherapy may boost the anti-tumor immune response in some patients, as suggested by the persisting ALK-antibody titers and observations of measurable anti-ALK T cell responses after—but not before—chemotherapy in a few patients [48,72]. Cyclophosphamide and anthracyclines are examples of chemotherapeutic drugs included in the classical ALCL chemotherapy strategies that lead to immunogenic cell death [73]. Among 54 chemotherapeutic drugs tested for their pharmacological effects on the maturation, survival, and growth of dendritic cells, the tubulin inhibitor vinblastine was shown to be the most potent inducer of DC maturation [74,75]. Thus, the observed efficacy of vinblastine monotherapy in patients with relapsed ALCL [27,76] might be due to dual action: its direct cytotoxic effect and its augmentation of the patients’ anti-tumor immunity by inducing the phenotypic and functional maturation of DCs. A low toxicity vinblastine monotherapy should, therefore, be studied for the front-line treatment of low-risk ALCL patients. In addition to the low risk of toxicity and late effects, this would allow for an outpatient treatment of ALCL patients. 

The classical immunotherapy in leukemia and lymphoma is allogeneic SCT. Its efficacy as a consolidation therapy for children with ALCL relapses has been well established [30,31,32,33]. Given the overwhelming indications for a strong graft versus ALCL effect, reduced intensity conditioning regimen should be developed for the allogeneic SCT against ALCL.

Since ALK-positive ALCLs express PD-L1 [66,67], immune checkpoint inhibitors, such as nivolumab or pembrolizumab, might increase the armamentarium of drugs for the treatment of ALCL. The observed efficacy of the PD1 inhibitor nivolumab for refractory disease in two patients highlights the possible clinical implications [77,78]. Whether this efficacy regarding remission induction translates into a cure must be evaluated in clinical trials.

More specific immunotherapies targeting ALCL surface markers or ALK include anti-CD30 chimeric antigen receptor (CAR) T cells, vaccination strategies against ALK, and possible ALK-specific T cell therapies. CD30-specific CAR T cells have been tested in mouse models and a phase I clinical trial so far [79]. One patient with relapsed ALK-positive ALCL reached remission after four doses of CD30-specific CAR T cells [80]. The possible therapeutic use of CAR T cells directed against the extracellular domain of ALK is being explored against ALK-expressing neuroblastoma [81]. ALK fusion proteins in ALCL, however, do not contain the transmembrane and extracellular domains and are expressed exclusively intracellularly. Therefore, ALK-directed CAR T cells are not a therapeutic option for ALK-positive ALCLs. 

Chiarle et al. showed the in vivo potential of a vaccination therapy with truncated ALK DNA, as well as this approach in combination with chemotherapy, in a mouse model [53]. The preclinical efficacy and existence of a “boostable” autologous response against ALK in humans call for the design of an ALK epitope-directed vaccination study in patients in remission after chemotherapy. The most suitable patients would be those with a pre-existing immune response, i.e., those with a low relapse risk suffering from late relapses. Patients with a very weak immune response against ALK usually relapse very early, within three months after therapy, which makes them less suitable for a vaccination approach. 

Tools for the selection and cultivation of antigen-specific T cells for adoptive immunotherapy have been established for several cancers [82]. However, whether ALK-specific T cells can be selected from patients and augmented for therapeutic use has not been studied yet. 

## 7. Conclusions

The available data suggest that immunotherapies targeting ALK have a high potential to revolutionize treatment strategies for ALK-positive ALCL in the future. One of the major challenges is the definition of the most suitable immunotherapy for an individual patient. It is currently unclear which group of patients might benefit from a vaccination or unspecific immune stimulation for the induction of life-long tumor control and which patients require an allogeneic SCT for cure. The deciphering of the host and tumor factors influencing the strength and efficacy of an autologous immune response against ALK-positive ALCL might help individual decision-making regarding consolidation therapy in the future.

Vaccination against ALK presumably has a very low safety risk and likely needs a pre-existent boostable immune response to be effective in the time before relapses occur. Vaccination studies could, therefore, be of benefit and should be studied for adults and children with low-risk ALCL. The focus for studies against very high risk or relapsed ALCL might include the optimization of allogeneic SCT and check-point inhibitors.

Both the cellular and humoral immune response in ALCL has become increasingly elucidated in the past years. However, more work is needed to further unravel the immunology of ALK-related malignancies. For the successful development of a vaccine, the antigenic ALK epitopes recognized by cells of the adaptive immune system need to be characterized. 

## References

[1] Falini B., Pileri S., Zinzani P.L., Carbone A., Zagonel V., Wolf-Peeters C., Verhoef G., Menestrina F., Todeschini G., Paulli M. (1999). ALK+ lymphoma: Clinico-pathological findings and outcome. Blood.

[2] Burkhardt B., Zimmermann M., Oschlies I., Niggli F., Mann G., Parwaresch R., Riehm H., Schrappe M., Reiter A., Group B.F.M. (2005). The impact of age and gender on biology, clinical features and treatment outcome of non-Hodgkin lymphoma in childhood and adolescence. Br. J. Haematol..

[3] Morris S.W., Kirstein M.N., Valentine M.B., Dittmer K.G., Shapiro D.N., Saltman D.L., Look A.T. (1994). Fusion of a kinase gene, ALK, to a nucleolar protein gene, npm, in non-Hodgkin’s lymphoma. Science.

[4] Perkins S.L., Pickering D., Lowe E.J., Zwick D., Abromowitch M., Davenport G., Cairo M.S., Sanger W.G. (2005). Childhood anaplastic large cell lymphoma has a high incidence of ALK gene rearrangement as determined by immunohistochemical staining and fluorescent in situ hybridisation: A genetic and pathological correlation. Br. J. Haematol..

[5] Damm-Welk C., Klapper W., Oschlies I., Gesk S., Rottgers S., Bradtke J., Siebert R., Reiter A., Woessmann W. (2009). Distribution of NPM1-ALK and x-ALK fusion transcripts in paediatric anaplastic large cell lymphoma: A molecular-histological correlation. Br. J. Haematol..

[6] Pulford K., Morris S.W., Turturro F. (2004). Anaplastic lymphoma kinase proteins in growth control and cancer. J. Cell. Physiol..

[7] Chiarle R., Voena C., Ambrogio C., Piva R., Inghirami G. (2008). The anaplastic lymphoma kinase in the pathogenesis of cancer. Nat. Rev. Cancer.

[8] Werner M.T., Zhao C., Zhang Q., Wasik M.A. (2017). Nucleophosmin-anaplastic lymphoma kinase: The ultimate oncogene and therapeutic target. Blood.

[9] Reiter A., Schrappe M., Tiemann M., Parwaresch R., Zimmermann M., Yakisan E., Dopfer R., Bucsky P., Mann G., Gadner H. (1994). Successful treatment strategy for ki-1 anaplastic large-cell lymphoma of childhood: A prospective analysis of 62 patients enrolled in three consecutive berlin-frankfurt-munster group studies. J. Clin. Oncol..

[10] Brugieres L., Deley M.C., Pacquement H., Meguerian-Bedoyan Z., Terrier-Lacombe M.J., Robert A., Pondarre C., Leverger G., Devalck C., Rodary C. (1998). Cd30(+) anaplastic large-cell lymphoma in children: Analysis of 82 patients enrolled in two consecutive studies of the French society of pediatric oncology. Blood.

[11] Seidemann K., Tiemann M., Schrappe M., Yakisan E., Simonitsch I., Janka-Schaub G., Dorffel W., Zimmermann M., Mann G., Gadner H. (2001). Short-pulse B-non-Hodgkin lymphoma-type chemotherapy is efficacious treatment for pediatric anaplastic large cell lymphoma: A report of the Berlin-Frankfurt-Munster group trial NHL-BFM 90. Blood.

[12] Williams D.M., Hobson R., Imeson J., Gerrard M., McCarthy K., Pinkerton C.R., United Kingdom Children’s Cancer Study Group (2002). Anaplastic large cell lymphoma in childhood: Analysis of 72 patients treated on the United Kingdom children’s cancer study group chemotherapy regimens. Br. J. Haematol..

[13] Mori T., Kiyokawa N., Shimada H., Miyauchi J., Fujimoto J. (2003). Anaplastic large cell lymphoma in Japanese children: Retrospective analysis of 34 patients diagnosed at the national research institute for child health and development. Br. J. Haematol..

[14] Rosolen A., Pillon M., Garaventa A., Burnelli R., d’Amore E.S., Giuliano M., Comis M., Cesaro S., Tettoni K., Moleti M.L. (2005). Anaplastic large cell lymphoma treated with a leukemia-like therapy: Report of the Italian association of pediatric hematology and oncology (aieop) LNH-92 protocol. Cancer.

[15] Lowe E.J., Sposto R., Perkins S.L., Gross T.G., Finlay J., Zwick D., Abromowitch M., Children’s Cancer Group Study (2009). Intensive chemotherapy for systemic anaplastic large cell lymphoma in children and adolescents: Final results of children’s cancer group study 5941. Pediatr. Blood Cancer.

[16] Brugieres L., Le Deley M.C., Rosolen A., Williams D., Horibe K., Wrobel G., Mann G., Zsiros J., Uyttebroeck A., Marky I. (2009). Impact of the methotrexate administration dose on the need for intrathecal treatment in children and adolescents with anaplastic large-cell lymphoma: Results of a randomized trial of the eicnhl group. J. Clin. Oncol..

[17] Alexander S., Kraveka J.M., Weitzman S., Lowe E., Smith L., Lynch J.C., Chang M., Kinney M.C., Perkins S.L., Laver J. (2014). Advanced stage anaplastic large cell lymphoma in children and adolescents: Results of ANHL0131, a randomized phase III trial of apo versus a modified regimen with vinblastine: A report from the children’s oncology group. Pediatr. Blood Cancer.

[18] Le Deley M.C., Rosolen A., Williams D.M., Horibe K., Wrobel G., Attarbaschi A., Zsiros J., Uyttebroeck A., Marky I.M., Lamant L. (2010). Vinblastine in children and adolescents with high-risk anaplastic large-cell lymphoma: Results of the randomized ALCL99-vinblastine trial. J. Clin. Oncol..

[19] Pro B., Advani R., Brice P., Bartlett N.L., Rosenblatt J.D., Illidge T., Matous J., Ramchandren R., Fanale M., Connors J.M. (2012). Brentuximab vedotin (SGN-35) in patients with relapsed or refractory systemic anaplastic large-cell lymphoma: Results of a phase ii study. J. Clin. Oncol..

[20] Pro B., Advani R., Brice P., Bartlett N.L., Rosenblatt J.D., Illidge T., Matous J., Ramchandren R., Fanale M., Connors J.M. (2017). Five-year results of brentuximab vedotin in patients with relapsed or refractory systemic anaplastic large cell lymphoma. Blood.

[21] Mosse Y.P., Lim M.S., Voss S.D., Wilner K., Ruffner K., Laliberte J., Rolland D., Balis F.M., Maris J.M., Weigel B.J. (2013). Safety and activity of crizotinib for paediatric patients with refractory solid tumours or anaplastic large-cell lymphoma: A children’s oncology group phase 1 consortium study. Lancet Oncol..

[22] Mosse Y.P., Voss S.D., Lim M.S., Rolland D., Minard C.G., Fox E., Adamson P., Wilner K., Blaney S.M., Weigel B.J. (2017). Targeting ALK with crizotinib in pediatric anaplastic large cell lymphoma and inflammatory myofibroblastic tumor: A children’s oncology group study. J. Clin. Oncol..

[23] Gambacorti Passerini C., Farina F., Stasia A., Redaelli S., Ceccon M., Mologni L., Messa C., Guerra L., Giudici G., Sala E. (2014). Crizotinib in advanced, chemoresistant anaplastic lymphoma kinase-positive lymphoma patients. J. Natl. Cancer Inst..

[24] Brugieres L., Quartier P., Le Deley M.C., Pacquement H., Perel Y., Bergeron C., Schmitt C., Landmann J., Patte C., Terrier-Lacombe M.J. (2000). Relapses of childhood anaplastic large-cell lymphoma: Treatment results in a series of 41 children—A report from the french society of pediatric oncology. Ann. Oncol..

[25] Mori T., Takimoto T., Katano N., Kikuchi A., Tabuchi K., Kobayashi R., Ayukawa H., Kumagai M.A., Horibe K., Tsurusawa M. (2006). Recurrent childhood anaplastic large cell lymphoma: A retrospective analysis of registered cases in Japan. Br. J. Haematol..

[26] Woessmann W., Zimmermann M., Lenhard M., Burkhardt B., Rossig C., Kremens B., Lang P., Attarbaschi A., Mann G., Oschlies I. (2011). Relapsed or refractory anaplastic large-cell lymphoma in children and adolescents after berlin-frankfurt-muenster (BFM)-type first-line therapy: A bfm-group study. J. Clin. Oncol..

[27] Ruf S., Brugieres L., Pillon M., Zimmermann M., Attarbaschi A., Mellgren K., Williams D., Uyttebroeck A., Wrobel G., Reiter A. (2015). Risk-adapted therapy for patients with relapsed or refractory ALCL—final report of the prospective ALCL-relapse trial of the eicnhl. Br. J. Haematol..

[28] Iwahara T., Fujimoto J., Wen D., Cupples R., Bucay N., Arakawa T., Mori S., Ratzkin B., Yamamoto T. (1997). Molecular characterization of ALK, a receptor tyrosine kinase expressed specifically in the nervous system. Oncogene.

[29] Woessmann W. (2018).

[30] Woessmann W., Peters C., Lenhard M., Burkhardt B., Sykora K.W., Dilloo D., Kremens B., Lang P., Fuhrer M., Kuhne T. (2006). Allogeneic haematopoietic stem cell transplantation in relapsed or refractory anaplastic large cell lymphoma of children and adolescents—A Berlin-Frankfurt-Munster group report. Br. J. Haematol..

[31] Gross T.G., Hale G.A., He W., Camitta B.M., Sanders J.E., Cairo M.S., Hayashi R.J., Termuhlen A.M., Zhang M.J., Davies S.M. (2010). Hematopoietic stem cell transplantation for refractory or recurrent non-Hodgkin lymphoma in children and adolescents. Biol. Blood Marrow Transplant..

[32] Strullu M., Thomas C., Le Deley M.C., Chevance A., Kanold J., Bertrand Y., Jubert C., Dalle J.H., Paillard C., Baruchel A. (2015). Hematopoietic stem cell transplantation in relapsed ALK+ anaplastic large cell lymphoma in children and adolescents: A study on behalf of the sfce and SFGM-TC. Bone Marrow Transplant..

[33] Fukano R., Mori T., Kobayashi R., Mitsui T., Fujita N., Iwasaki F., Suzumiya J., Chin M., Goto H., Takahashi Y. (2015). Haematopoietic stem cell transplantation for relapsed or refractory anaplastic large cell lymphoma: A study of children and adolescents in Japan. Br. J. Haematol..

[34] Lamant L., McCarthy K., d’Amore E., Klapper W., Nakagawa A., Fraga M., Maldyk J., Simonitsch-Klupp I., Oschlies I., Delsol G. (2011). Prognostic impact of morphologic and phenotypic features of childhood ALK-positive anaplastic large-cell lymphoma: Results of the ALCL99 study. J. Clin. Oncol..

[35] Abramov D., Oschlies I., Zimmermann M., Konovalov D., Damm-Welk C., Wossmann W., Klapper W. (2013). Expression of CD8 is associated with non-common type morphology and outcome in pediatric anaplastic lymphoma kinase-positive anaplastic large cell lymphoma. Haematologica.

[36] Damm-Welk C., Busch K., Burkhardt B., Schieferstein J., Viehmann S., Oschlies I., Klapper W., Zimmermann M., Harbott J., Reiter A. (2007). Prognostic significance of circulating tumor cells in bone marrow or peripheral blood as detected by qualitative and quantitative pcr in pediatric NPM-ALK-positive anaplastic large-cell lymphoma. Blood.

[37] Savan R., McFarland A.P., Reynolds D.A., Feigenbaum L., Ramakrishnan K., Karwan M., Shirota H., Klinman D.M., Dunleavy K., Pittaluga S. (2011). A novel role for IL-22R1 as a driver of inflammation. Blood.

[38] Mellgren K., Hedegaard C.J., Schmiegelow K., Muller K. (2012). Plasma cytokine profiles at diagnosis in pediatric patients with non-Hodgkin lymphoma. J. Pediatr. Hematol. Oncol..

[39] Knorr F., Damm-Welk C., Ruf S., Singh V.K., Zimmermann M., Reiter A., Woessmann W. (2017). Blood cytokine concentrations of pediatric anaplastic lymphoma kinase-positive anaplastic large cell lymphoma patients. Haematologica.

[40] Pasqualini C., Minard-Colin V., Saada V., Lamant L., Delsol G., Patte C., Le Deley M.C., Valteau-Couanet D., Brugieres L. (2014). Clinical analysis and prognostic significance of haemophagocytic lymphohistiocytosis-associated anaplastic large cell lymphoma in children. Br. J. Haematol..

[41] Wu J., Li X., Song W., Fang Y., Yu L., Liu S., Churilov L.P., Zhang F. (2017). The roles and applications of autoantibodies in progression, diagnosis, treatment and prognosis of human malignant tumours. Autoimmun. Rev..

[42] Zaenker P., Gray E.S., Ziman M.R. (2016). Autoantibody production in cancer—The humoral immune response toward autologous antigens in cancer patients. Autoimmun. Rev..

[43] Tsou P., Katayama H., Ostrin E.J., Hanash S.M. (2016). The emerging role of b cells in tumor immunity. Cancer Res..

[44] Pulford K., Falini B., Banham A.H., Codrington D., Roberton H., Hatton C., Mason D.Y. (2000). Immune response to the ALK oncogenic tyrosine kinase in patients with anaplastic large-cell lymphoma. Blood.

[45] Ait-Tahar K., Cerundolo V., Banham A.H., Hatton C., Blanchard T., Kusec R., Becker M., Smith G.L., Pulford K. (2006). B and CTL responses to the ALK protein in patients with ALK-positive ALCL. Int. J. Cancer.

[46] Mussolin L., Bonvini P., Ait-Tahar K., Pillon M., Tridello G., Buffardi S., Lombardi A., Pulford K., Rosolen A. (2009). Kinetics of humoral response to ALK and its relationship with minimal residual disease in pediatric ALCL. Leukemia.

[47] Ait-Tahar K., Damm-Welk C., Burkhardt B., Zimmermann M., Klapper W., Reiter A., Pulford K., Woessmann W. (2010). Correlation of the autoantibody response to the ALK oncoantigen in pediatric anaplastic lymphoma kinase-positive anaplastic large cell lymphoma with tumor dissemination and relapse risk. Blood.

[48] Mussolin L., Pillon M., Zimmermann M., Carraro E., Basso G., Knoerr F., Woessmann W., Damm-Welk C. (2017). Course of anti-ALK antibody titres during chemotherapy in children with anaplastic large cell lymphoma. Br. J. Haematol..

[49] Mussolin L., Damm-Welk C., Pillon M., Zimmermann M., Franceschetto G., Pulford K., Reiter A., Rosolen A., Woessmann W. (2013). Use of minimal disseminated disease and immunity to NPM-ALK antigen to stratify ALK-positive ALCL patients with different prognosis. Leukemia.

[50] Damm-Welk C., Siddiqi F., Fischer M., Hero B., Narayanan V., Camidge D.R., Harris M., Burke A., Lehrnbecher T., Pulford K. (2016). Anti-ALK antibodies in patients with ALK-positive malignancies not expressing NPM-ALK. J. Cancer.

[51] Awad M.M., Mastini C., Blasco R.B., Mologni L., Voena C., Mussolin L., Mach S.L., Adeni A.E., Lydon C.A., Sholl L.M. (2017). Epitope mapping of spontaneous autoantibodies to anaplastic lymphoma kinase (ALK) in non-small cell lung cancer. Oncotarget.

[52] Montgomery R.B., Makary E., Schiffman K., Goodell V., Disis M.L. (2005). Endogenous anti-HER2 antibodies block HER2 phosphorylation and signaling through extracellular signal-regulated kinase. Cancer Res..

[53] Chiarle R., Martinengo C., Mastini C., Ambrogio C., D’Escamard V., Forni G., Inghirami G. (2008). The anaplastic lymphoma kinase is an effective oncoantigen for lymphoma vaccination. Nat. Med..

[54] Passoni L., Scardino A., Bertazzoli C., Gallo B., Coluccia A.M., Lemonnier F.A., Kosmatopoulos K., Gambacorti-Passerini C. (2002). ALK as a novel lymphoma-associated tumor antigen: Identification of 2 HLA-A2.1-restricted CD8^+^ T-cell epitopes. Blood.

[55] Passoni L., Gallo B., Biganzoli E., Stefanoni R., Massimino M., Di Nicola M., Gianni A.M., Gambacorti-Passerini C. (2006). In vivo t-cell immune response against anaplastic lymphoma kinase in patients with anaplastic large cell lymphomas. Haematologica.

[56] Beckhove P., Feuerer M., Dolenc M., Schuetz F., Choi C., Sommerfeldt N., Schwendemann J., Ehlert K., Altevogt P., Bastert G. (2004). Specifically activated memory T cell subsets from cancer patients recognize and reject xenotransplanted autologous tumors. J. Clin. Investig..

[57] Enamorado M., Iborra S., Priego E., Cueto F.J., Quintana J.A., Martinez-Cano S., Mejias-Perez E., Esteban M., Melero I., Hidalgo A. (2017). Enhanced anti-tumour immunity requires the interplay between resident and circulating memory CD8(+) t cells. Nat. Commun..

[58] Nizard M., Roussel H., Diniz M.O., Karaki S., Tran T., Voron T., Dransart E., Sandoval F., Riquet M., Rance B. (2017). Induction of resident memory T cells enhances the efficacy of cancer vaccine. Nat. Commun..

[59] Voena C., Menotti M., Mastini C., Di Giacomo F., Longo D.L., Castella B., Merlo M.E.B., Ambrogio C., Wang Q., Minero V.G. (2015). Efficacy of a cancer vaccine against ALK-rearranged lung tumors. Cancer Immunol. Res..

[60] Singh K.V., Werner S., Hackstein H., Lennerz V., Reiter A., Wolfel T., Damm-Welk C., Woessmann W. (2016). Analysis of nucleophosmin-anaplastic lymphoma kinase (NPM-ALK)-reactive CD8(+) t cell responses in children with NPM-ALK(+) anaplastic large cell lymphoma. Clin. Exp. Immunol..

[61] Janssen E.M., Lemmens E.E., Wolfe T., Christen U., von Herrath M.G., Schoenberger S.P. (2003). Cd4+ t cells are required for secondary expansion and memory in CD8^+^ T lymphocytes. Nature.

[62] Crotty S. (2015). A brief history of t cell help to b cells. Nat. Rev. Immunol..

[63] Ait-Tahar K., Barnardo M.C., Pulford K. (2007). CD4 T-helper responses to the anaplastic lymphoma kinase (ALK) protein in patients with ALK-positive anaplastic large-cell lymphoma. Cancer Res..

[64] Chen D.S., Mellman I. (2013). Oncology meets immunology: The cancer-immunity cycle. Immunity.

[65] Motz G.T., Coukos G. (2013). Deciphering and reversing tumor immune suppression. Immunity.

[66] Marzec M., Zhang Q., Goradia A., Raghunath P.N., Liu X., Paessler M., Wang H.Y., Wysocka M., Cheng M., Ruggeri B.A. (2008). Oncogenic kinase NPM/ALK induces through STAT3 expression of immunosuppressive protein CD274 (PD-L1, B7-H1). Proc. Natl. Acad. Sci. USA.

[67] Andorsky D.J., Yamada R.E., Said J., Pinkus G.S., Betting D.J., Timmerman J.M. (2011). Programmed death ligand 1 is expressed by non-Hodgkin lymphomas and inhibits the activity of tumor-associated T cells. Clin. Cancer Res..

[68] Kasprzycka M., Marzec M., Liu X., Zhang Q., Wasik M.A. (2006). Nucleophosmin/anaplastic lymphoma kinase (NPM/ALK) oncoprotein induces the t regulatory cell phenotype by activating stat3. Proc. Natl. Acad. Sci. USA.

[69] Thibodeau J., Bourgeois-Daigneault M.C., Huppe G., Tremblay J., Aumont A., Houde M., Bartee E., Brunet A., Gauvreau M.E., de Gassart A. (2008). Interleukin-10-induced march1 mediates intracellular sequestration of mhc class ii in monocytes. Eur. J. Immunol..

[70] Chattopadhyay G., Shevach E.M. (2013). Antigen-specific induced t regulatory cells impair dendritic cell function via an IL-10/march1-dependent mechanism. J. Immunol..

[71] Tze L.E., Horikawa K., Domaschenz H., Howard D.R., Roots C.M., Rigby R.J., Way D.A., Ohmura-Hoshino M., Ishido S., Andoniou C.E. (2011). CD83 increases mhc ii and CD86 on dendritic cells by opposing IL-10-driven march1-mediated ubiquitination and degradation. J. Exp. Med..

[72] Singh V.K. (2018).

[73] Zitvogel L., Kepp O., Kroemer G. (2011). Immune parameters affecting the efficacy of chemotherapeutic regimens. Nat. Rev. Clin. Oncol..

[74] Tanaka H., Matsushima H., Mizumoto N., Takashima A. (2009). Classification of chemotherapeutic agents based on their differential in vitro effects on dendritic cells. Cancer Res..

[75] Tanaka H., Matsushima H., Nishibu A., Clausen B.E., Takashima A. (2009). Dual therapeutic efficacy of vinblastine as a unique chemotherapeutic agent capable of inducing dendritic cell maturation. Cancer Res..

[76] Brugieres L., Pacquement H., Le Deley M.C., Leverger G., Lutz P., Paillard C., Baruchel A., Frappaz D., Nelken B., Lamant L. (2009). Single-drug vinblastine as salvage treatment for refractory or relapsed anaplastic large-cell lymphoma: A report from the french society of pediatric oncology. J. Clin. Oncol..

[77] Hebart H., Lang P., Woessmann W. (2016). Nivolumab for refractory anaplastic large cell lymphoma: A case report. Ann. Intern Med..

[78] Rigaud C., Abbou S., Minard-Colin V., Geoerger B., Scoazec J.Y., Vassal G., Jaff N., Heuberger L., Valteau-Couanet D., Brugieres L. (2017). Efficacy of nivolumab in a patient with systemic refractory ALK+ anaplastic large cell lymphoma. Pediatr. Blood Cancer.

[79] Hombach A.A., Gorgens A., Chmielewski M., Murke F., Kimpel J., Giebel B., Abken H. (2016). Superior therapeutic index in lymphoma therapy: CD30(+) CD34(+) hematopoietic stem cells resist a chimeric antigen receptor T-cell attack. Mol. Ther..

[80] Ramos C.A., Ballard B., Zhang H., Dakhova O., Gee A.P., Mei Z., Bilgi M., Wu M.F., Liu H., Grilley B. (2017). Clinical and immunological responses after CD30-specific chimeric antigen receptor-redirected lymphocytes. J. Clin. Investig..

[81] Walker A.J., Majzner R.G., Zhang L., Wanhainen K., Long A.H., Nguyen S.M., Lopomo P., Vigny M., Fry T.J., Orentas R.J. (2017). Tumor antigen and receptor densities regulate efficacy of a chimeric antigen receptor targeting anaplastic lymphoma kinase. Mol. Ther..

[82] Maus M.V., Fraietta J.A., Levine B.L., Kalos M., Zhao Y., June C.H. (2014). Adoptive immunotherapy for cancer or viruses. Annu. Rev. Immunol..

